# The accuracy of the Italian version of the Hypomania Checklist (HCL-32) for the screening of bipolar disorders and comparison with the Mood Disorder Questionnaire (MDQ) in a clinical sample

**DOI:** 10.1186/1745-0179-2-2

**Published:** 2006-03-08

**Authors:** Mauro Giovanni Carta, Maria Carolina Hardoy, Mariangela Cadeddu, Andrea Murru, Andrea Campus, Pier Luigi Morosini, Alex Gamma, Jules Angst

**Affiliations:** 1 Division of Psychiatry, Department of Public Health, University of Cagliari, Cagliari, Italy; 2 National Institute of Health, Rome, Italy; 3 Zurich University Psychiatric Hospital, Psychiat. Uni. Klinik, PO Box 68, Lenggstrasse 31, CH-8029, Zurich, Switzerland

## Abstract

**Background:**

The study measured the accuracy of the Italian version of the Hypomania Checklist (HCL-32) for self-assessment as a screening instrument for bipolar disorder (BPD) in a psychiatric setting and compared results with a previous study, carried out in a comparable sample and in the same setting, using the Mood Disorder Questionnaire (MDQ).

**Methods:**

123 consecutive subjects attending a psychiatric division were screened for BPD using the Italian translation of the HCL-32, and diagnostically interviewed with the SCID by physicians. The sample of the previous study using the MDQ consisted of 154 subjects.

**Results:**

On the basis of the SCID: 26 received a diagnosis of bipolar/schizoaffective disorder, 57 were diagnosed as having at least another psychiatric disorder in Axis-I, whilst 40 were unaffected by any type of psychiatric disorder. Comparing the bipolar with all other patients the HCL-32 showed a good accuracy: cut-off 8: sensitivity 0.92-specificity 0.48; cut-off 10: sensitivity 0.88-specificity 0.54; cut-off 12: sensitivity 0.85-specificity 0.61. The accuracy for BPD-II (10) remains good: cut-off 8: sensitivity 0.90-specificity 0.42; cut-off 10: sensitivity 0.80-specificity 0.47; cut-off 12: sensitivity 0.80-specificity 0.54. The comparison with the MDQ performance shows that both screening tools may show good results, but HCL-32 seems to be more sensitive in detecting BPD-II.

**Conclusion:**

Our results seem to indicate good accuracy of HCL-32 as a screening instrument for BPD in a psychiatric setting, with a low rate of false negatives, and a fairly good degree of identification of BPD-II.

## Background

Bipolar disorder is a recurring psychiatric condition, of chronic nature and highly invalidating. It is often under-diagnosed, thus delaying the administration of efficient treatments; it has been estimated that as many as one to two thirds of individuals with bipolar disorder do not receive appropriate treatment due to misdiagnosis [1]. Particularly hypomania as an element of bipolar-II disorder is very often not experienced and recognized by the subject as pathological, therefore not reported to doctors and under-diagnosed in 25 to 50% of depressive patients [2].

Thus an easily administered screening instrument for self-assessment may be useful in clinical practice, particularly in settings in which a great number of medical patients are referred for psychiatric screening before medical treatments, such as interferon, hormone therapies with a burden of risk for psychiatric disorders.

The aim of the present study, carried out in a sample of psychiatric patients attending a mental health clinic, with an elevated proportion of patients coming from the general hospital for psychiatric evaluation, was to obtain a preliminary standardization of one Italian version of the HCL-32 as a screening instrument for bipolar disorders, [2] and to compare results with a previous study, carried out in a comparable sample and the same setting, using the Mood Disorder Questionnaire (MDQ) [3]. The MDQ is a internationally recognized screening instrument for bipolar disorders [4-6] (Hirschfeld et al. 2000, 2003a, 2003b) that seems to be sensitive for identifying bipolar-I disorders but probably less so for bipolar disorders-II [7].

## Methods

### Study design

The study design consisted in the evaluation of the accuracy of one Italian version of HCL-32 [2], using the Structured Clinical Interview for DSM-IV Axis-I Disorders (SCID) [8] as a gold standard. The results were compared with the findings of a previous study, carried out in the same setting and with the same methodology, in which the Mood Disorder Questionnaire (MDQ) was applied.

The SCID was administered by physicians working in the field of psychiatry for at least three years, all of whom had undergone specific training for the use of the SCID.

Immediately prior to the interview all subjects had filled in the Italian version of the HCL-32. Written informed consent was obtained from all study participants. The questionnaire had been translated into Italian before the start of the research project, had been back-translated into English, and approval had been obtained from one of the authors of the original version.

### Sample

A consecutive series of 123 subjects (41 males, mean age 36.6 ± 11.0 years; 81 females, mean age 37.9 ± 12.4 years) referred to the Division of Psychiatry of the University of Cagliari (Italy). The subjects were either (a) seeking psychiatric care, (b) had been referred by a general medical hospital for psychiatric evaluation before receiving medical treatments involving an elevated psychiatric risk (interferon, hormone therapy, etc.), or (c) were applying for legal certification of their mental capacities (for driving and/or gun licences, etc). The sample of the previous study using the MDQ consisted of 154 subjects (61 males, mean age 35.9 ± 12.3 years; 93 females, mean age 38.4 ± 12.5 years) also referred consecutively to the Division of Psychiatry of the University of Cagliari (Italy).

### Instruments

The Structured Clinical Interview for DSM-IV Disorders of Axis-I [8] is a semi-structured interview aimed at formulating the main diagnoses covered by Axis-I of DSM-IV [9].

The HCL-32 is a self-administered paper and pencil inventory made up of 32 yes/no items used to identify the hypomanic component in patients with depressive episodes in order to help the clinician to diagnose bipolar-II and minor bipolar disorders in psychiatric and general medical practice [2]. In order to help diagnose hypomania the instrument is designed to assess the personal and social role consequences of hypomanic symptoms. It also takes into account the subject's current overall affective status (low – as usual – high) as a potentially interfering variable in answering the questions. Several questions also have the potential to reveal the extent to which the patient has insight into his condition, which is important for treatment considerations [2].

We evaluated the discriminatory capacity (patients with a diagnosis of bipolar I or II disorder or schizoaffective bipolar type versus patients with other psychiatric diagnoses or with no psychiatric diagnosis, according to the findings of the SCID) of all 32 items of the HCL-32. A specific evaluation concerning the accuracy in detecting bipolar II disorders was also carried out.

The accuracy of the HCL-32 was calculated in terms of sensitivity and specificity for each theoretically possible cut-off point (number of positive answers). Overall performance of the scale was graphically assessed by means of the Receiver Operating Characteristic Analysis [10].

## Results

**Figure 1:**
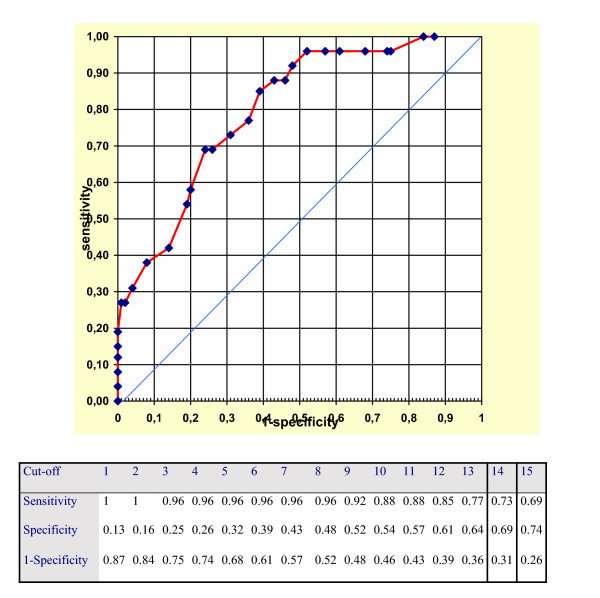
ROC analysis of the performance of the HCL-32 in the total sample.

Table 1 shows the psychiatric diagnoses in Axis-I based on the SCID. Of all subjects interviewed (total N = 123): 26 (21.14%) received a diagnosis of bipolar or schizoaffective bipolar disorder, 57 (58.76%) were diagnosed as having at least one psychiatric disorder in Axis-I (other than bipolar or schizoaffective), whilst 40 (41.24%) were unaffected by any type of psychiatric disorder. Of the 26 patients identified as "bipolar cases" at the SCID interview 14 (53.8%) had bipolar I disorder, 10 (38.5%) had bipolar II disorder and 2 (7.8%) schizoaffective bipolar disorder. Figure 1 illustrates the performance of the HCL-32 by means of ROC analysis and reports for each cut-off sensitivity and specificity, comparing the bipolar with all other patients. Table 2 compares the best performing cut-off scores of the HCL-32 for bipolar I and bipolar II disorder with the results of the Mood Disorder Questionnaire recently validated by our group in the same setting [3].

**Table 1:** Psychiatric diagnoses in Axis-I formulated according to findings of the SCID semi-structured interview [8].

PSYCHIATRIC DIAGNOSES IN AXIS-I						
Bipolar or Schizoaffective disorder		At least another 1 disorder in Axis-I		No diagnosis		Total subjects

N	%	N	%	N	%	N

26	21.14	57	58.76	40	41.24	123

**Table 2:** Best performing cut-off of HCL-32 and MDQ for bipolar II disorders.

MDQ Cut-off	MDQ sensitivity	MDQ specificity	HCL-32 Cut-off	HCL-32 sensitivity	HCL-32 specificity
Total Sample					
4	0.90	0.56	8	0.92	0.48
5	0.84	0.70	10	0.88	0.54
6	0.76	0.86	12	0.85	0.61
Bipolar II disorders					
4	0.80	0.45	8	0.90	0.42
5	0.70	0.55	10	0.80	0.47
6	0.55	0.65	12	0.80	0.54

## Discussion

The results of this study seem to indicate a fairly good performance of the HCL-32, at least from a preliminary point of view, in a specialist setting applying a traditional gold standard (SCID diagnoses).

Results confirm the preliminary validation conducted on an Italian and a Swedish sample where HCL-32 distinguished between bipolar and major depressive out-patients. That result was based on the hypothesis that DSM-IV criteria for hypomania are too strict [11]. For this reason the previous study used a modified version of SCID with its diagnoses as the gold standard.

Our study used a naturalistic sample of patients referred to the Division of Psychiatry of the University of Cagliari (Italy) for psychiatric treatment or legal certification. The use of different cut-off points depends on the intended use of the scale. For two-stage investigations (in which it is important that "cases" are recognized during the screening phase because all positive cases will subsequently undergo clinical assessment), it is necessary to reduce the "false negatives" to a bare minimum. It is therefore preferable to use a very high sensitivity, even though this may prejudice the specificity. Cut-off point 4 of HCL-32 (sensitivity 0.90, specificity 0.58) would for example allow an accurate two-stage investigation to be carried out, whilst reducing interviews by more than 50%. It should moreover be taken into account that epidemiologic studies are performed in a specific setting (general population, general practitioners) where the proportion of psychiatric disorders is obviously lower than that observed in a clinical sample recruited from a University Division of Psychiatry. The latter would reasonably lead us to presume that the screening instrument would perform better. For a one-stage study in a general psychiatry setting the ideal cut-off point could be 12 (sensitivity 0.85, specificity 0.61). The latter is the nearest point to the 0.1 vertex of the ROC diagram. Indeed, this point represents the ideal screener (sensitivity 1, specificity 1). On the other hand, the area depicted by the curve beyond the diagonal of the diagram represents the area of validity of the screener. It should moreover be underlined that the HCL-32 is a simple and easy-to-use tool. The comparison with the MDQ performance shows that both screening tools may show good results, but HCL-32 seems to be more sensitive in detecting bipolar II disorders. Cut-offs with same sensitivity (0.80, at cut-off 4 for MDQ, and at cut-off 12 for HCL-32) show a better specificity for HCL-32 (0.45 for MDQ, 0.54 for HCL-32); cut-offs showing the same specificity (cut-off 5 for MDQ = 0.55 and cut-off 12 HCL-32 = 0.54) have better sensitivity for HCL-32 (0.80 versus 0.70).

An important problem is the recognition of bipolar-II disorders in patients with major depressive episodes; thus the HCL-32 for the self-assessment of hypomanic symptoms might be very helpful for identifying suspected and manifest cases of bipolar disorders.

Further studies are needed to verify the accuracy of this tool in non-psychiatric settings and in the general population.

## Competing interests

The author(s) declare that they have no competing interests.
